# Correlation of ADIPOQ Gene Single Nucleotide Polymorphisms with Bone Strength Index in Middle-Aged and the Elderly of Guangxi Mulam Ethnic Group

**DOI:** 10.3390/ijerph182413034

**Published:** 2021-12-10

**Authors:** Min Zhou, Ning Ning, Yueming Jiang, Michael Aschner, Xiufeng Huang, Xiaoyun Bin, Jinhua Wang

**Affiliations:** 1 School of Public Health and Management, Youjiang Medical University for Nationalities, Baise 533000, China; 00365@ymun.edu.cn; 2 School of Public Health, Shantou University Medical College, Shantou 515000, China; 20nning@stu.edu.cn; 3 Department of Toxicology, School of Public Health, Guangxi Medical University, Nanning 530021, China; ymjiang@gxmu.edu.cn; 4 Department of Molecular Pharmacology, Albert Einstein College of Medicine, Bronx, NY 10461, USA; michael.aschner@einsteinmed.org; 5 School of Basic Medical Sciences, Youjiang Medical University for Nationalities, Baise 533000, China; 00113@ymun.edu.cn

**Keywords:** Mulam, middle-aged and elderly, osteoporosis, bone strength index, ADIPOQ, polymorphism

## Abstract

Background: Osteoporosis (OP) is a common orthopedic disease in the elderly, and Adiponectin (ADIPOQ) is closely related to bone metabolism. Objective: To determine the relationship between five single nucleotide polymorphism (SNP) loci in the ADIPOQ gene and osteoporosis in middle-aged and elderly Mulam subjects in Hechi, Guangxi. Methods: This case-control study included 297 middle-aged and elderly Mulam subjects with normal bone mass, 49 subjects with reduced bone mass, and 38 subjects with osteoporosis. Five loci (*rs266729*, *rs1063539*, *rs2241766*, *rs3774261*, *rs710445*) of the ADIPOQ in the Mulam subjects were genotyped using SNP with multiple-base extension. Results: The bone strength index (SI) of middle-aged and elderly Mulam subjects showed an overall decreasing trend when the subjects were older. Age, muscle mass, and subcutaneous fat content were the main factors influencing the SI in Mulam subjects. The GC genotype of *rs266729* and the GA and GG genotypes of *rs710445* were significantly correlated with risk of bone loss (*p* < 0.05). *rs2241766* and *rs1063539* showed strong LD (D’ > 0.8, r^2^ > 0.33). *rs710445* and rs266729 loci and *rs3774261* and *rs2241766* loci showed complete LD (D’ = 1). Conclusions: The GC genotype at *rs266729* of the ADIPOQ gene, the GA and GG genotypes at *rs710445*, and the haplotypes CCGAA and GGTAG correlated with osteoporosis (*p* < 0.05). The allele C of *rs1063539*, *rs266729* and *rs710445* may afford protection for osteoporosis. The allele G may be the genetic susceptibility gene for osteoporosis, increasing the risk of osteoporosis.

## 1. Introduction

Osteoporosis (OP) is a common bone disease in middle-aged and elderly populations. The disease is characterized by decreased bone mass, damaged bone tissue microstructure, increased bone brittleness and propensity for fractures. Adiponectin (APN), also known as ACRP30, APM1 and ADIPOQ, is an endogenous bioactive polypeptide, a protein secreted by adipocytes. The gene is located in chromosome 3Q27 and consists of three exons and two introns [1]. It has anti-inflammatory, anti-atherosclerosis, and anti-platelet aggregation properties, to name a few. Adiponectin, expressed by adipocytes, has anti-inflammatory, anti-atherosclerosis, anti-platelet aggregation and other biological effects, and it is associated with diabetes mellitus, coronary heart disease, metabolic syndrome and other diseases [2,3]. The effect of adiponectin on bone metabolism is mediated by three distinct pathways, namely, (1) an autocrine or paracrine pathway; (2) an endocrine pathway; and (3) an insulin signaling pathway [4]. At present, there is no clear-cut evidence on the relationship between adiponectin and bone metabolism. In recent years, it has been posited that adiponectin is closely related to bone metabolism [5,6,7]. Several studies have shown that adiponectin can promote bone formation, increasing bone mass by inhibiting the growth of osteoclasts [8,9,10]. In contrast, other studies have shown that adiponectin levels are negatively correlated with bone mineral density [11,12,13]. In addition, adiponectin has shown no significant correlation with bone mineral density [14].

Guangxi is a multi-ethnic autonomous region, with Han, Zhuang, Miao and Mulam ethnic groups in this area. Luocheng is the only Mulam minority autonomous county in China. The prevalence of hypertension, dyslipidemia and diabetes among Mulam subjects is higher than the national level [15]. Compared with the Yao, Miao and Maonan ethnic groups, Mulam women have the lowest bone mineral density, the highest prevalence rate of osteoporosis, and the highest percentage of bone mass reduction [16]. Factors affecting etiology include geographical environment, diet, genetics and other factors [17]; therefore, the prevalence of osteoporosis varies among ethnic groups. The study of adiponectin gene polymorphism in middle-aged and elderly Mulam subjects is of great significance, as it offers insight to pathogenesis of osteoporosis, the distribution characteristics of pathogenic and susceptible genes; hence, it can shed novel empirical data on osteoporosis etiology, diagnosis and treatment.

## 2. Objects and Methods

### 2.1. Objects

From January to March 2021, 384 middle-aged and elderly members (45 years old and above) of the Mulam ethnic minority (for a minimum of 3 generations) were randomly selected as the research subjects. Among them, 165 were males (average age 63.6 ± 9.78), and 219 were females (mean age 60.16 ± 10.14). They were divided into 6 groups, each group covering 5 years (for example, 45–50, 50–55, etc.). Based on the diagnostic criteria [18], 297 patients with normal bone mass were designated as the control group, and 49 patients with reduced bone mass and 38 patients with osteoporosis were assigned to the reduced bone mass case group. All participants gave informed written consent, and the study was approved by the ethics committee of the university.

Inclusion: middle-aged and elderly members (45 years old and above), Mulam ethnic minority (for a minimum of 3 generations). Exclusion: hypertension, diabetes, heart disease, liver and renal insufficiency, ovariectomy, thyroid, parathyroid, endocrine, blood system, connective tissue disease and medications affecting bone metabolism within 1 year.

### 2.2. Methods

#### 2.2.1. Bone Strength Index Determination

Achilles Express (GE, Fairfield, IA, USA) was used to measure the bone strength index, after inputting the subject’s name, height, age, gender and other relevant data. Both the inside and outside of the right heel were disinfected and placed inside the instrument to measure the bone strength index.

#### 2.2.2. Body Composition Index Determination

Body composition and BMI were measured with a bio-electrical impedance analyzer (TANITA, MC-180, Tokyo, Japan).

#### 2.2.3. APN Gene SNP Genotyping

Adiponectin 5 SNP loci were genotyped in 384 samples using the imLDRTM multiple SNP genotyping kit. (1) Extraction of genomic DNA of leukocytes: 3 mL venous blood was removed, and sodium citrate was used for anticoagulant treatment of the blood. DNA was extracted by phenol chloroform method. (2) Primer design and PCR amplification: Primer design was carried out after the conserved sequence of the gene was analyzed and integrated through NCBI database. Obtained primers were evaluated by Oligo software. The obtained primer sequences were sent to Shanghai Sangon Company for synthesis. The primers are detailed in Supplementary Materials Table S1. PCR products containing SNP sites were obtained with Qiagen’s Hotstar Taq multiplex PCR. The PCR product was purified by shrimp alkali enzyme (SAP) (Promega Inc., Singapore) and Exonuclease I enzyme (Epicentre Inc., Madison, WI, USA), and the ligase detection reaction was performed with the imLDRTM multiple SNP typing kit of Shanghai Tianhao Biological Co., Ltd. (Shanghai, China). The resulting ligation products were analyzed by a ABI3730XL sequencer. The raw data collected on the ABI3730XL sequencer was analyzed using GeneMapper 4.1 (AppliedBiosystems, Waltham, MA, USA).

### 2.3. Statistical Analysis

SPSS 23.0 was used to process the data. One-sample Kolmogorov–Smirnov and QQ plots were used to test whether the data conformed to a normal distribution. After testing, the data conformed to a normal distribution. First, descriptive analysis was conducted for the data. T test, chi square test and analysis of variance (ANOVA) were conducted for comparison between two or more groups. The bone strength index was used as the dependent variable for correlation analysis with all body composition indexes, and then the multivariate stepwise regression analysis was performed on the variables with statistical significance in the correlation analysis. *p* < 0.05 was considered to be statistically significant. Broken line graph was used to analyze changes in bone strength index with age. Chi-square test was used to determine whether the polymorphic genotypes met Hardy–Weinberg equilibrium. The linkage disequilibrium parameters D’, r^2^ between two or more groups and comparison of haploid frequency were analyzed with SHesis online software (http://analysis.bio-x.cn, accessed on 10 May 2021).

## 3. Results

### 3.1. Trends of Bone Strength Index of the Mulam Middle-Aged and Elderly People Growing with Age

**Figure 1:**
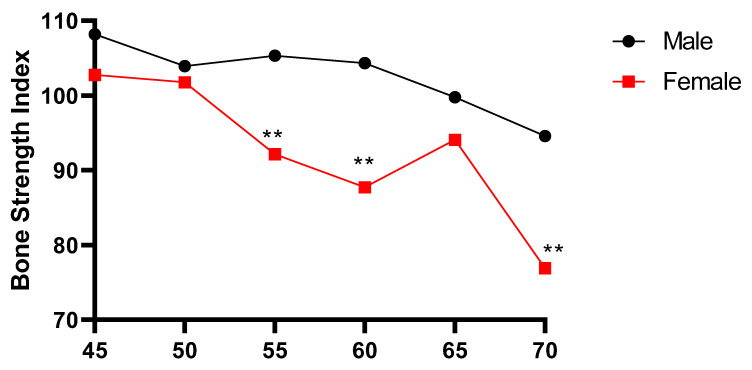
Trends of bone SI with age in middle-aged and elderly Mulam people. (male vs. female in the same age group, ** *p* < 0.01).

**Figure 2:**
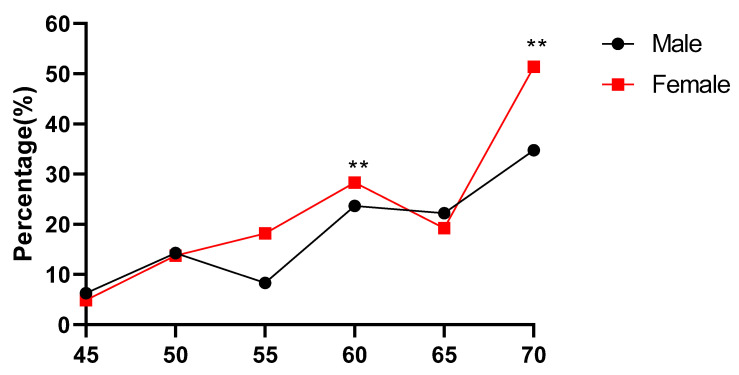
Trends of percentage changes with age in individuals with bone loss among middle-aged and elderly Mulam people. (Comparison of prevalence rates between male and female of the same age group, ** *p* < 0.01).

As shown in Table 1 and Figure 1 and Figure 2, the bone strength index (SI) of the Mulam ethnic minority middle-aged and elderly subjects generally showed a decreasing trend with the age increasing, and the difference of SI among male multiple age groups was statistically significant (*p* < 0.01). The SI of 45-year-old group was higher than that of 70-year-old group, and the comparative difference of SI between the 70-year-old group and the other 5 groups was statistically significant (*p* < 0.05). There was no significant difference in the SI between the other groups. The difference in SI among female multiple age groups was also statistically significant (*p* < 0.01), with that of the 45-year-old group being higher than those of most age groups, except for the 50-year-old group. In addition, there were also statistical differences between men and women in the 55-year-old group, 60-year-old group and 70-year-old group (*p* < 0.01), and the SI in women was lower than that of men in all groups. As shown in Figure 2, the prevalence of Osteopenia in the middle-aged and elderly of the Mulam ethnic minority increased with increased age. There was a statistically significant difference in the prevalence rate of osteopenia between the 60-year-old group and the 70-year-old group (*p*< 0.01), indicating that the prevalence rate of women was higher than that of men. The decrease in the prevalence rate in the 65-year-old group may be affected by the relatively small sample size.

### 3.2. Analysis of Factors Influencing on Bone Strength INDEX of Middle-Aged and Elderly People of the Mulam Ethnic Minority

Pearson correlation analysis showed that the SI of the middle-aged and elderly people of the Mulam ethnic minority was positively correlated with height, weight and muscle mass, and was negatively correlated with age, fat content and subcutaneous fat content (Table 2). Set control group = 0 and case group = 1. Taking SI as the dependent variable, and height, weight, muscle mass, age, fat mass and subcutaneous fat content as independent variables, multiple stepwise regression analysis was conducted. The results showed that age, muscle mass and subcutaneous fat content were the main factors influencing on SI in the middle-aged and elderly Mulam people (Table 3).

### 3.3. Frequency Distribution of ADIPOQ Genotypes and Alleles in the Middle-Aged and Elderly Mulam People

SNP loci of the ADIPOQ gene were analyzed, establishing the existence of six loci polymorphisms on the ADIPOQ gene (*rs266729*, *rs1063539*, *rs2241766*, *rs3774261*, *rs710445*) in the Mulam ethnic minority in Hechi area. χ^2^ test showed that all of the samples complied with the Hardy–Weinberg genetic equilibrium law (*p* > 0.05). The GC genotype at the *rs266729* locus was significantly correlated with the risk of osteopenia (OR = 1.934, *p* = 0.009), with the difference between allele G and C at the *rs1063539* locus being statistically significant. In addition, the GA and GG genotypes at the *rs710445* locus were also correlated with risk for osteopenia (GA: OR = 1.799, *p* = 0.043, GG: OR = 2.109, *p* = 0.028). The difference between allele A and G was statistically significant. There was no significant difference in the frequency distribution of alleles and other genotypes (Table 4). In addition, in Supplementary Tables S2 and S3, we show the comparison of BMI and gender with the genotype and allelic gene frequency distribution of ADIPOQ, and the results showed that there was no difference between ADIPOQ and BMI and gender in Mulam population.

### 3.4. Analysis of Linkage Unbalance among ADIPOQ Gene Polymorphism Loci in the Middle-Aged and Elderly Mulam People

**Figure 3:**
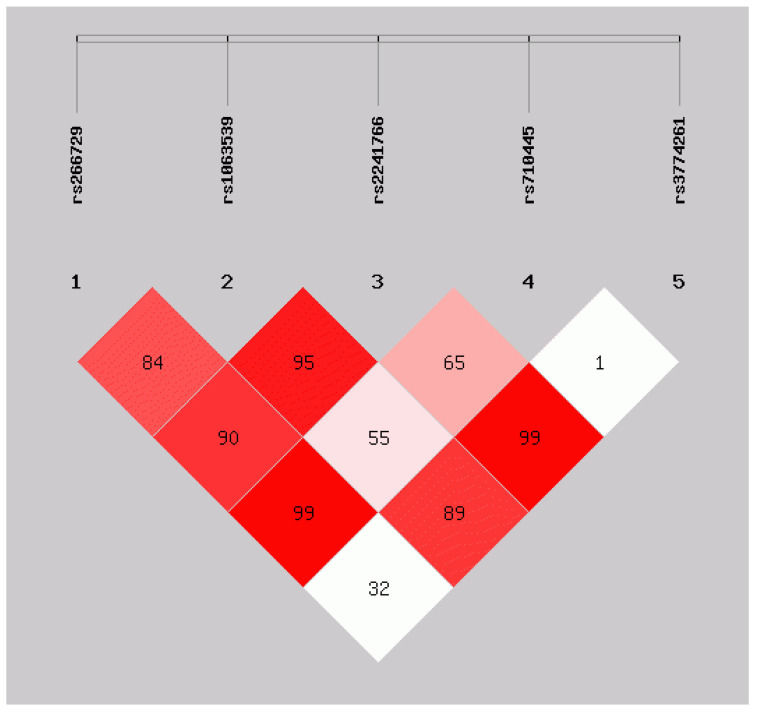
ADIPOQ gene linkage disequilibrium and haplotype block structure. Value in cells indicate logarithm of odds (LOD)score of D’.

Online analysis by SHEsis showed that *rs2241766* loci and *rs1063539* loci showed strong linkage disequilibrium (LD) (D’ > 0.8, r^2^ > 0.33). As shown in Figure 3, there was complete LD at *rs710445* and *rs266729* and *rs3774261* and *rs2241766* (D’ = 1). Seven haplotypes were produced. Carriers of the CCGAA gene haplotype in the case group had a lower risk of developing the disease than those in the control group (OR = 0.639, 95% CI = 0.418–0.979, *p* = 0.038). GGTAG gene haploid carriers in the case group had an increased risk of disease compared with the control group (OR = 2.182, 95% CI = 1.274–3.738, *p* = 0.004). The distribution of all haploid types in the case group and the control group was statistically significant (χ^2^ = 12.80, *p* = 0.046) (Table 5).

## 4. Discussion

Hechi is the main settlement of Mulam ethnic group. Studying the genetic polymorphism of Mulam ethnic group is of great significance for exploring the distribution characteristics of pathogenic genes and susceptible genes of osteoporosis, studying the pathogenesis of osteoporosis, and diagnosing and treating at the genetic level. Through the study of ADIPOQ gene loci and osteoporosis risk, the ADIPOQ gene has been proven to be a potential useful genetic marker for predicting the risk of osteoporosis in middle-aged and elderly Mulam people.

### 4.1. Changes in Bone Strength Index

Bone strength index (SI) correlates with bone mineral density (BMD) and bone strength and is the best predictor of risk of bone fracture [19]. The results of this study showed that the bone strength index of middle-aged and elderly Mulam ethnic minority decreased with age and that the incidence of bone loss was generally increased with the increase of age. In some age groups, bone strength index in women was significantly lower than in men, and the incidence of osteopenia was higher in females vs. men, consistent with results of a published meta-analysis [20]. After menopause, estrogen level drops, and osteoclastic activity in females is significantly greater than osteogenesis, resulting in decreased bone mass, and increased bone fragility. The significant difference in bone strength index between men and women may also reflect the fact that middle-aged and elderly men of the Mulam ethnic minority continue to engage in farming and other physical activities, even at advanced age. These activities stimulate muscle contraction, producing pressure load on local bones and thus delaying the age-related decline in bone strength.

### 4.2. Analysis of Risk Factors for Osteoporosis

Body composition includes fat-free weight and fat mass. Fat-free weight is composed of bone mass and muscle mass, while fat mass is composed of subcutaneous fat mass and visceral fat mass. The relationship between body composition and bone strength is also different in different regions and different races. Multiple stepwise regression showed that muscle mass was a protective factor for bone strength, while age and subcutaneous fat content were risk factors for bone strength. Akune’s [21] study has shown that adipocytes and osteoblasts contain homologous bone marrow progenitor cells. Activation of peroxisome proliferation-activated receptor γ promotes the differentiation of bone marrow progenitors into adipocytes and inhibits the formation of osteoblasts, which may be related to the fact that subcutaneous fat mass is a risk factor for bone strength index in middle-aged and elderly Mulam ethnic minority. The research shows that physical exercise is considered an effective means to stimulate bone osteogenesis in osteoporotic patients [22]. Therefore, increasing exercise, increasing muscle mass, and controlling subcutaneous fat content are helpful in increasing bone strength index and preventing osteoporosis in middle-aged and elderly Mulam ethnic minority.

### 4.3. Relationship between SNP and Bone Strength Index

Wang Jinhua et al. [7,23] found that the *rs1063539* polymorphism in the adiponectin gene correlated with bone mineral density in women of Zhuang nationality in Guangxi, and the *rs3774261* polymorphism correlated with bone mineral density in men of Zhuang nationality. No correlation was found between *rs266729* and *rs710445* polymorphisms and bone mineral density in Zhuang nationality, contrasting with the results in the present study. A bone mineral density survey of 907 healthy postmenopausal women in South Korea showed that *rs1501299* was associated with spinal fracture, and subjects with GG genotype had the highest incidence of spinal fracture [24]. Kim et al. [25] showed that the *rs16850799* and *rs34010966* polymorphisms of adiponectin receptor 1 (Adipor1) were significantly associated with femoral neck bone mineral density. The association between *rs34010966* and bone mineral density has shown a gene x dose interaction.

The novel results of this study suggested that the frequency distribution of *rs1063539*, *rs266729* and *rs710445* alleles of the ADIPOQ gene was statistically significant different between the case and control groups, suggesting that allele C may reduce the risk of osteopenia, and allele G may increase its risk. The results of genetic haplotype analysis suggested that haploid CCGAA may reduce the risk of osteopenia, and haploid GGTAG may increase its risk. Individuals with haploid GGTAG had a 2.182-fold increased risk of osteopenia compared with those without the haploid GGTAG.

This study showed that *rs1063539*, *rs266729*, and *rs710445* genetic polymorphisms were associated with disease phenotypes, and there were differences from the result in the Zhuang population mentioned above. These loci may be caused by linkage disequilibrium between gene polymorphism loci and disease loci in Mulam population. Whether this locus shows the same results in different ethnic groups and larger sample size needs further study. The results also showed that there was no difference in genotype and gene frequency of *rs2241766* and *rs3774261* in the Mulam osteoporosis population and may be through interaction with other sites, mutual influence and synergistic pathogenesis. Its own disease does not play the role of main pathogenic factor and has nothing to do with osteoporosis.

In summary, osteoporosis is a common disease with multiple factors, and genetic factors play an important role in it. This study found that GC genotypes at the *rs266729* locus of ADIPOQ gene, GA and GG genotype at *rs710445* locus, and haploid CCGAA and GGTAG were correlated with osteoporosis. Among them, allele C may be a protective factor in the etiology of osteoporosis, while allele G may be an inherited susceptibility gene for osteoporosis, increasing the risk of the disease. The exact molecular mechanism of ADIPOQ gene polymorphism and osteoporosis remains unclear and could be profitable in future studies.

## Figures and Tables

**Table 1:** Result of bone strength index.

Age Groups	Bone Strength Index			
	n	Males	n	Females
45-	16	108.19 ± 16.41	41	102.76 ± 13.35
50-	14	103.93 ± 19.76	29	101.76 ± 14.25
55-	24	105.33 ± 12.03	33	92.15 ± 17.89 ^ab^
60-	38	104.32 ± 16.24	53	87.72 ± 13.36 ^ab^
65-	27	99.78 ± 12.89	26	94.08 ± 18.50 ^a^
70-	46	94.59 ± 15.92 ^a^	37	76.89 ± 15.2 ^ab^
F value	3.125		14.747	
*p*	0.01		<0.01	

^a^ Compared to the 45-year-old group, *p* < 0.01; ^b^ male vs. female in the same age group, *p* < 0.01.

**Table 2:** Correlation analysis of bone strength index with each index.

Variables	r	*p*
Age	−0.343	<0.01
Height	0.182	<0.01
Weight	0.123	0.016
BMI (kg/m^2^)	0.023	0.654
Fat content	−0.108	0.035
Muscle mass	0.245	<0.01
Waist-to-hip ratio	−0.064	0.212
Visceral fat content	−0.061	0.235
Subcutaneous fat content	−0.118	0.021

**Table 3:** Multiple linear regression analysis of bone strength index and partial index.

Variables	B	S.E.	β	t	*p*
Age	−0.613	0.084	−0.344	−7.318	<0.01
Muscle mass	0.584	0.123	0.220	4.733	<0.01
Subcutaneous fat content	−0.590	0.184	−0.151	−3.203	<0.01

**Table 4:** Genotype and allelic gene frequency distribution in five SNPs of ADIPOQ.

Loci	Genotype	Normal	Osteopenia	OR (95% CI)	χ^2^	*p*
*rs266729*	CC	176	38	1.000		-
	GC	103	43	1.934 (1.173–3.186)	6.807	0.009
	GG	18	6	1.544 (0.575–4.147)	0.347	0.556
Allelic gene						
	G	139	55	1.000		-
	C	455	119	0.661 (0.456–0.959)	4.803	0.028
*rs1063539*	CC	27	4	1.000		-
	GC	144	35	1.641 (0.539–4.994)	0.773	0.379
	GG	126	48	2.571 (0.855–7.736)	2.996	0.083
Allelic gene						
	G	396	131	1.000		-
	C	198	43	0.656 (0.447–0.964)	4.645	0.031
*rs2241766*	GG	26	3	1.000		-
	GT	136	36	2.294 (0.657–8.009)	1.778	0.182
	TT	135	48	3.081 (0.892–10.645)	3.457	0.063
Allelic gene						
	G	188	42	1.000		-
	T	406	132	1.455 (0.987–2.145)	3.62	0.057
*rs3774261*	AA	89	24	1.000		-
	GA	152	49	1.034 (0.734–1.455)	0.036	0.85
	GG	56	14	0.87 (0.458–1.651)	0.182	0.67
Allelic gene						
	A	330	97	1.000		-
	G	264	77	0.992 (0.706–1.394)	0.002	0.964
*rs710445*	AA	116	22	1.000		-
	GA	126	43	1.799 (1.015–3.189)	4.109	0.043
	GG	55	22	2.109 (1.077–4.132)	4.843	0.028
Allelic gene						
	A	358	87	1.000		-
	G	236	87	1.517 (1.080–2.130)	5.824	0.016

**Table 5:** Haplotype frequency distribution of ADIPOQ polymorphisms.

Haplotype	Case	Control	*p*	OR (95% CI)
CCGAA	0.188	0.262	0.038	0.639 (0.418–0.979)
CCGAG	0.032	0.037	0.745	0.855 (0.331–2.208)
CGTAA	0.063	0.071	0.691	0.870 (0.437–1.732)
CGTAG	0.114	0.089	0.345	1.302 (0.752–2.255)
CGTGA	0.231	0.257	0.451	0.857 (0.573–1.281)
GGTAG	0.138	0.068	0.004	2.182 (1.274–3.738)
GGTGG	0.172	0.146	0.421	1.207 (0.763–1.907)

## Data Availability

The data presented in this study are available on request from the corresponding author. The data are not publicly available due to the privacy of the participants.

## Supplementary Materials

The following are available online at https://www.mdpi.com/article/10.3390/ijerph182413034/s1, Table S1: Primer sequences of ADIPOQ gene used in polymerase chain reaction, Table S2. Genotype and allelic gene frequency distribution in five SNPs of ADIPOQ (Grouped by BMI), Table S3. Genotype and allelic gene frequency distribution in five SNPs of ADIPOQ (Grouped by sex).

## Abbreviations

OP	osteoporosis
SI	bone strength index
APN	adiponectin
BMD	bone mineral density
LD	linkage disequilibrium
