# Supplementary material for: Correlation of ADIPOQ Gene Single Nucleotide Polymorphisms with Bone Strength Index in Middle-Aged and the Elderly of Guangxi Mulam Ethnic Group
Source: Int J Environ Res Public Health. 2021 Dec 10;18(24):13034. doi: 10.3390/ijerph182413034 (PMC8701406; doi:10.3390/ijerph182413034)
Supplement: Supplementary file 1 [file ijerph-18-13034-s001.zip › ijerph-1445700-supplementary.pdf]

Table S1. Primer sequences of ADIPOQ gene used in polymerase chain reaction

Loci	Forward primer	Extend primer
rs266729	GGCTCTGTGTGGACTGTGGAGA	GGCCTAGAAGCAGCCTGGAGAA
rs1063539	GTTGGGGTGGGCTCCTTACAGA	CCTCAGTTTCTCTCAGGCCTCTTTTG
rs2241766	GATGCTGTTGCTGGGAGCTGTT	TTCTCACCAGGGGTGCCATCTC
rs3774261	ACACCCCCTGAAAGGCTCAGTC	ACCCTGCCAGCCCTTTATGTG
rs710445	TCAAGAGGTTCTAATGAGGCCAGC	GGCGAGCAGGATTAGGCTTCAG

Table S2. Genotype and allelic gene frequency distribution in five SNPs of ADIPOQ (Grouped by BMI).

Loci	Genotype	Obesity	Overweight	normal	underweight	χ^2	P
rs266729	CC	20	85	103	6		
	GC	12	44	87	3	4. 702	0. 195
	GG	3	10	10	1	0. 569	0. 904
Allelic gene							
	G	18	64	107	5		
	C	52	214	293	15	1. 217	0. 749
rs1063539	CC	1	12	16	2		
	GC	15	72	88	4	2. 613	0. 455
	GG	19	55	96	4	3. 614	0. 306
Allelic gene							
	G	53	182	280	12		
	C	17	96	120	8	3. 963	0. 265
rs2241766	GG	1	10	16	2		
	GT	13	70	85	4	2. 743	0. 433
	TT	21	59	99	4	3. 561	0. 313
Allelic gene							
	G	15	90	117	8		
	T	55	188	283	12	4. 258	0. 235
rs3774261	AA	9	46	55	3		
	GA	17	74	103	7	0. 559	0. 906
	GG	9	19	42	0	5. 779	0. 105
Allelic gene							
	A	35	166	213	13		
	G	35	112	187	7	4. 404	0. 221
rs710445	AA	13	49	70	6		
	GA	16	62	89	2	3. 004	0. 391
	GG	6	28	41	2	0. 626	0. 890
Allelic gene							
	A	42	160	229	14		
	G	28	118	171	6	1. 411	0. 703

Table S3. Genotype and allelic gene frequency distribution in five SNPs of ADIPOQ (Grouped by sex).

Loci	Genotype	male	female	χ^2	P
rs266729	CC	90	124		
	GC	67	79	0. 519	0. 471
	GG	8	16	0. 678	0. 410
Allelic gene					
	G	83	111		
	C	247	327	0. 004	0. 952
rs1063539	CC	14	17		
	GC	75	104	0. 115	0. 734
	GG	76	98	0. 024	0. 878
Allelic gene					
	G	227	300		
	C	103	138	0. 008	0. 931
rs2241766	GG	16	13		
	GT	69	103	2. 305	0. 129
	TT	80	103	1. 326	0. 250
Allelic gene					
	G	101	129		
	T	229	309	0. 119	0. 730
rs3774261	AA	43	70		
	GA	90	111	1. 339	0. 247
	GG	32	38	1. 049	0. 306
Allelic gene					
	A	176	251		
	G	154	187	1. 203	0. 273
rs710445	AA	62	76		
	GA	70	99	0. 381	0. 537
	GG	33	44	0. 086	0. 769
Allelic gene					
	A	194	251		
	G	136	187	0. 170	0. 680
